# Synchronous double primary hepatic cancer consisting of hepatocellular carcinoma and cholangiolocellular carcinoma: a case report

**DOI:** 10.1186/s13256-018-1762-4

**Published:** 2018-08-18

**Authors:** Masateru Yamamoto, Akihiko Oshita, Takashi Nishisaka, Hideki Nakahara, Toshiyuki Itamoto

**Affiliations:** 1 Department of Gastroenterological, Breast and Transplant Surgery, Hiroshima Prefectural Hospital, Hiroshima, Japan; 2 Department of Gastroenterological and Transplant Surgery, Applied Life Science, Institute of Biomedical and Health Science, Hiroshima University, Hiroshima, Japan; 3 Department of Pathology Clinical Laboratory, Hiroshima Prefectural Hospital, Hiroshima, Japan

**Keywords:** Cholangiolocellular carcinoma, Chronic liver disease, Double primary hepatic cancer, Hepatocellular carcinoma, Synchronous

## Abstract

**Background:**

The incidence of synchronous double primary hepatic cancers is extremely low. Cholangiolocellular carcinoma is also a rare disease.

**Case presentation:**

A 58-year-old Japanese man was referred to our hospital for the treatment of multiple liver tumors revealed on computed tomography scans. He was hepatitis B and C positive and had undergone hemodialysis for 9 years due to chronic renal failure. Computed tomography scans revealed two hepatic tumors (each ≤ 1.0 cm in diameter) in segments 3 and 7. The preoperative diagnosis was multiple hepatocellular carcinomas. He underwent partial resections of his liver. The resected specimens revealed that the tumors in segments 3 and 7 were well-defined lesions of 8.0 mm and 14.0 mm, respectively. Pathological and immunohistochemical examinations confirmed the tumor in segment 3 to be a cholangiolocellular carcinoma and the tumor in segment 7 to be a hepatocellular carcinoma. Chronic inflammation could contribute to the different types of primary hepatic cancers. It may also give rise to various combinations of synchronous double primary hepatic cancer in patients with chronic liver disease.

**Conclusions:**

We describe the sixth case of synchronous double primary hepatic cancers consisting of hepatocellular carcinoma and cholangiolocellular carcinoma in chronic damaged liver and review the literature. In patients with chronic liver disease, careful surveillance with imaging studies should be mandatory as various types of primary hepatic cancers could develop.

## Background

Cholangiolocellular carcinoma (CoCC) is a rare disease that accounts for 0.6−1.0% of primary liver cancers [1, 2]. CoCC is classified as combined hepatocellular carcinoma (HCC) and cholangiocarcinoma with stem cell features according to the modified fourth edition of the World Health Organization classification system [3]. CoCC is associated with distinct clinical and histopathological characteristics.

Synchronous double primary hepatic cancer consisting of HCC and intrahepatic cholangiocarcinoma (ICC) is also rarely encountered in clinical practice. It accounts for 0.5–0.7% of primary liver cancers [4]. Only five cases of synchronous double primary hepatic cancer consisting of HCC and CoCC have been reported [5–9]. Here we describe the sixth case of the rare combination of double hepatic cancer and review the literature.

## Case presentation

A 58-year-old Japanese man was referred to our hospital for surgical treatment of two hepatic tumors. He had a history of blood transfusion at the age of 6 years during surgical treatment for a traumatic left femoral fracture. He was diagnosed as hepatitis B and C viral infection positive at the age of 30 years, and he had a history of interferon therapy at the age of 33 years. He also had a history of diabetes, and hemodialysis was introduced for diabetic renal failure at the age of 49 years. He had no familial history. Medical check-ups included computed tomography (CT) scans at his previous hospital each year. A CT scan revealed two hepatic tumors, and he was referred to our hospital 1 month later. His abdomen was soft and flat without ascites; his liver and spleen were not palpable in the subcostal area on physical examination. Laboratory findings on admission to our hospital included: platelet and white blood cell counts of 4.0 × 10^4^/μL and 2000/μL, respectively; hemoglobin, albumin, and total bilirubin levels of 12.0 g/dL, 3.8 g/dL, and 0.4 mg/dL, respectively; and aspartate and alanine aminotransferase, alkaline phosphatase, and gamma-glutamyl transpeptidase concentrations of 27 U/L, 27 U/L, 199 U/L, and 29 U/L, respectively. He had a prothrombin time (percent) of 66.5%. His Child–Pugh grade was corresponding to A. His indocyanine green retention rate at 15 minutes was 4.9%. Hepatitis B virus antigen and hepatitis C antibody were positive. His serum alpha-fetoprotein was elevated (126.0 ng/mL). The protein level induced by the vitamin K antagonist (18.0 mAU/mL) was within normal reference limits.

**Fig. 1:**
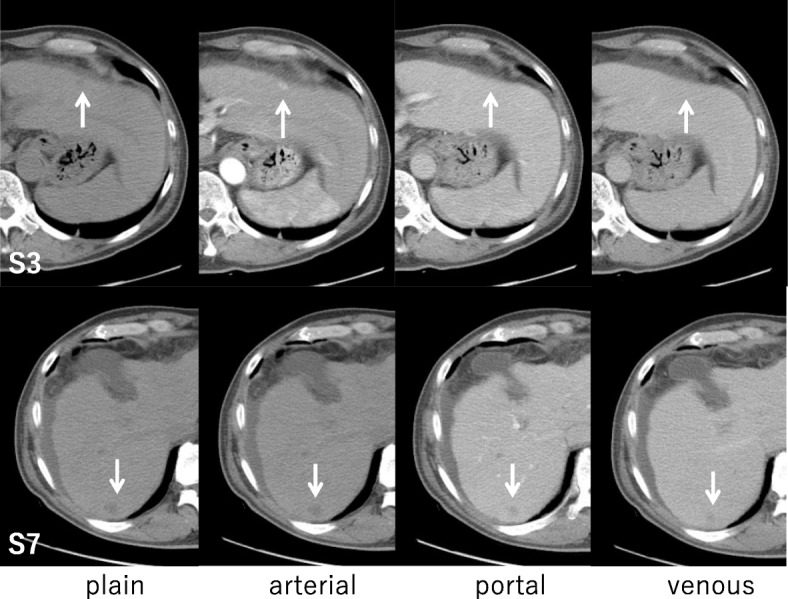
Abdominal computed tomography findings. Pre-contrast computed tomography scans revealed two hypoattenuating hepatic lesions (*arrows*; each ≤1.0 cm in diameter) in segments 3 and 7. The tumor in segment 3 was enhanced in the arterial phase and became isodense to liver parenchyma in the portal and venous phase. The tumor in segment 7 was not enhanced in any phase

**Fig. 2:**
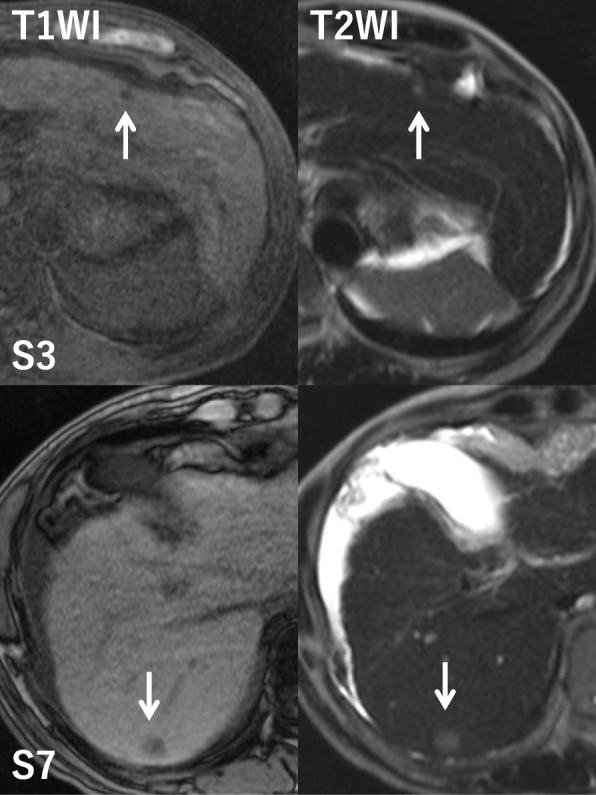
Abdominal magnetic resonance imaging. Magnetic resonance imaging revealed similar findings of low signal intensity on T1-weighted images and high signal intensity on T2-weighted images for both tumors (*arrows*). *T1WI* T1-weighted image, *T2WI* T2-weighted image

**Fig. 3:**
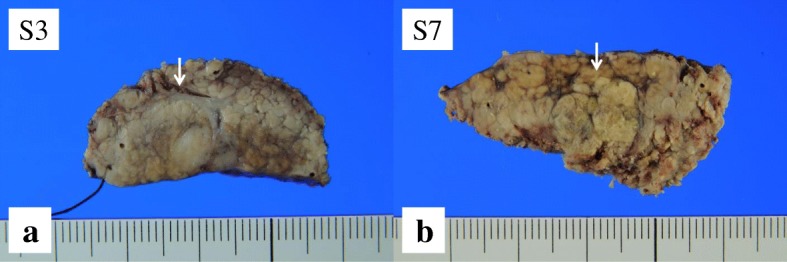
Surgically resected tumor tissue specimens. **a** The tumor in segment 3 was a well-defined lesion of 8.0 mm; **b** the tumor in segment 7 was a well-defined lesion of 14.0 mm

**Fig. 4:**
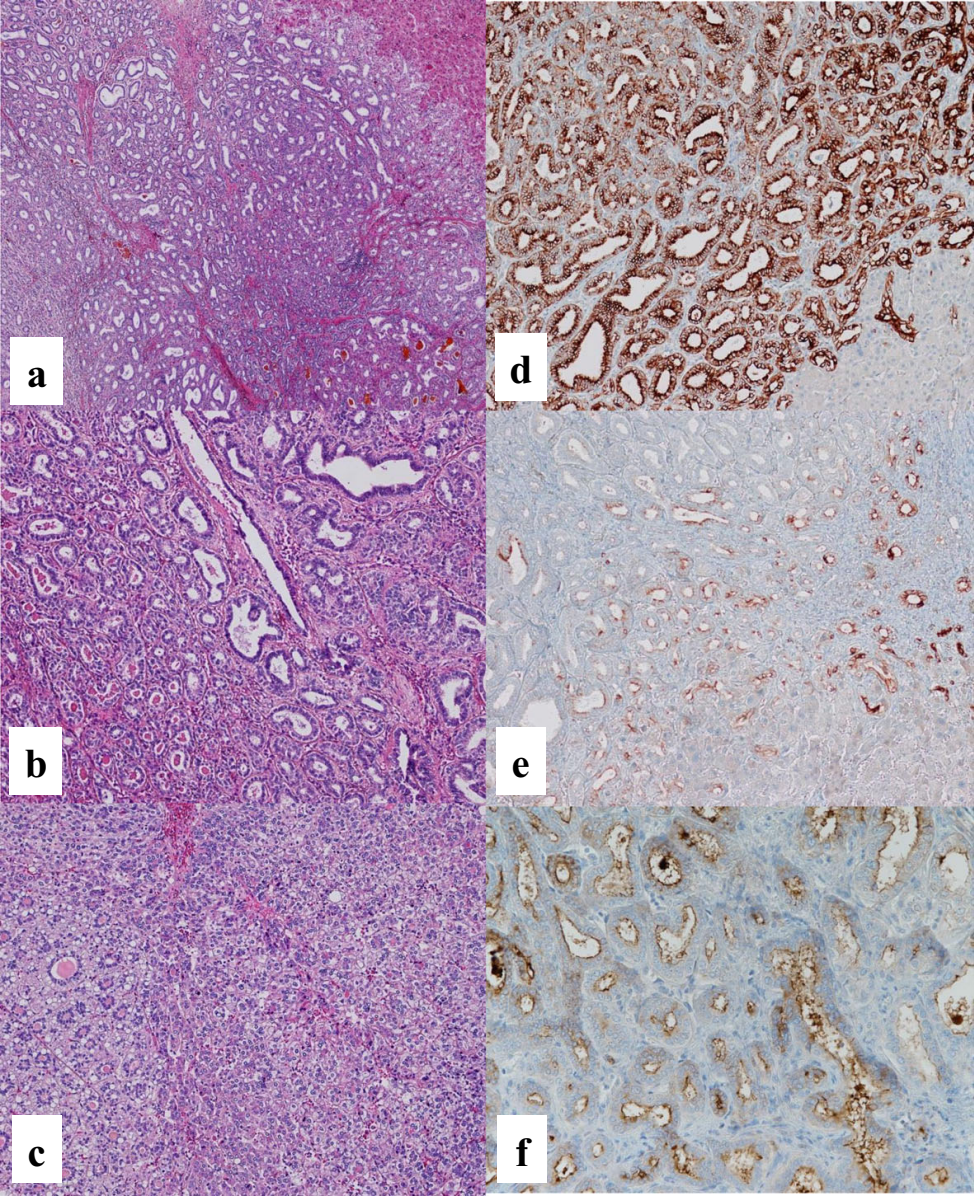
The pathological findings. **a** Tumor cells proliferated in an anastomosing pattern of Hering’s canal-like small glands with an abundant fibrous stroma that replaced the non-cancerous tissue in segment 3 (hematoxylin and eosin staining, × 100). **b** Small, regular, oval-shaped cells with mild atypia forming the luminal structure were observed in the segment 3 tumor (hematoxylin and eosin staining, × 200). **c** The tumor in segment 7 was partially surrounded by a thin fibrous capsule and septum. Atypical tumor cells proliferated in a trabecular and pseudoglandular pattern corresponding to moderately differentiated hepatocellular carcinoma (hematoxylin and eosin staining, × 100). On immunohistochemical analysis, segment 3 tumor cells stained positive for **d** cytokeratin 7 (× 200) and **e** cytokeratin 19 (× 200). **f** The membranous region of the lumen of the segment 3 tumor stained positive for epithelial membrane antigen (× 100)

Pre-contrast CT scans revealed two hypoattenuating hepatic lesions (each ≤1.0 cm in diameter) in segments 3 (S3) and 7 (S7). Contrast-enhanced CT scans revealed that the tumor in S3 was enhanced in the arterial phase and became isodense to liver parenchyma in the portal and venous phase. The tumor in S7 was not enhanced in any phase (Fig. 1). Magnetic resonance imaging revealed similar findings of low signal intensity on T1-weighted images and high signal intensity on T2-weighted images for both the S3 and S7 lesions (Fig. 2). The preoperative diagnosis was multiple HCCs. However, CT findings were not typical of HCCs. Partial resections of S3 and S7 were performed. The resected specimens revealed that the tumors in S3 and S7 were well-defined lesions of 8.0 mm and 14.0 mm, respectively (Fig. 3a, b). His preoperative platelet count was low, but a transfusion was not required. Pathological examination of tissue from the S3 tumor revealed small, regular, oval-shaped cells with mild atypia, which formed the luminal structure. Tumor cells proliferated in an anastomosing pattern of Hering’s canal-like small glands with an abundant fibrous stroma that replaced the non-cancerous tissue (Fig. 4a, b). The pathological diagnosis was CoCC. The tumor in S7 was partially surrounded by a thin fibrous capsule and septum. Atypical tumor cells proliferated in a trabecular and pseudoglandular pattern corresponding to moderately differentiated HCC (Fig. 4c). The pathological findings of each cancer resulted in T1aN0M0 stage I according to Union for International Cancer Control the 8th edition. The pathological findings of the non-cancerous tissue included liver cirrhosis. On immunohistochemical analysis, S3 tumor cells stained positive for cytokeratin 7 and cytokeratin 19 (Fig. 4d, e) and negative for hepatocyte paraffin 1 and Alcian blue. The membranous region of the lumen stained positive for epithelial membrane antigen (EMA; Fig. 4f).

His postoperative course was uneventful and he was discharged on postoperative day 13. He was alive without recurrence 36 months after surgery.

## Discussion

CoCC was first described by Steiner and Higginson [1] in 1959. CoCC is supposed to originate from the cholangioles (canals of Hering), where hepatic stem/progenitor cells exist [10]. It has been speculated that CoCC may be the malignant counterpart of these hepatic stem/progenitor cells. CoCCs occasionally have a HCC or ICC component that suggests they may have originated from hepatic stem/progenitor cells [11]. Recently, CoCC was classified as a subtype of combined HCC and ICC, although the lesion was classified as a distinct type of ICC [12]. CoCC tumor cells are small, regular, oval-shaped structures that have an abundant fibrous stroma and small, narrow lumen, which resemble cholangioles and the canals of Hering. In our case, a pathological diagnosis of CoCC was confirmed in the S3 by the characteristic findings outlined above and membranous positive staining for EMA. The usefulness of this staining pattern for the recognition of CoCC, normal cholangiole, or hepatic progenitor cells has been reported previously [11, 13].

With regard to imaging studies for CoCC, dynamic CT demonstrates enhancement on the arterial phase and homogeneous isodensity or low density on the venous phase, or peripheral enhancement on the arterial phase and central enhancement on the venous phase. The former characteristic resembles that of HCC and the latter characteristic resembles that of ICC. In the present case, the tumor in segment 3 was enhanced on the arterial phase and was faint on the venous phase; it was similar to HCC [5].

The biological behavior of CoCC remains elusive. CoCC exhibits similar features to those of HCC, including high incidences of hepatitis B and/or C virus infections and a frequent association with chronic liver disease [11, 13]. Patients with CoCC who have undergone a hepatectomy have a better prognosis than those with ICC [13], suggesting that the lower invasiveness of tumor cells into the vasculature surrounding the CoCC, with replacing growth patterns, results in a better prognosis [11]. CoCCs of > 4.0 cm in diameter are associated with higher recurrence rates than smaller CoCCs [11]. Further investigation of the prognostic significance of CoCC in a larger study population is required.

Five cases of synchronous double primary hepatic cancer consisting of HCC and CoCC have been reported previously [5–9] (Table 1). To the best of our knowledge, our case is the sixth case. Although a previous study [9] reviewed six cases of synchronous double primary hepatic cancer consisting of HCC and CoCC, the fifth case was excluded from our report because the patient had synchronous double primary hepatic cancer consisting of HCC and ICC, with a CoCC component that only occupied 20.0% of the tumor [14]. The above six cases, including our own, had a mean age of 63 (range, 45−71) years. A male predominance was evident, with five of the six cases associated with a hepatitis B and/or C virus infection. All of the patients had chronic liver disease, including one incidence of non-alcoholic steatohepatitis. Two types of tumors in four of the six patients exhibited a bilobar distribution in the liver. The mean size of the HCCs and CoCCs were 24.0 (range, 14.0−44.0) mm and 13.0 (range, 8.0−22.0) mm, respectively. The CoCC was smaller than the HCC in five of the six patients. In two cases, including our own, both types of tumor were < 2.0 cm in diameter, which is defined as small liver cancer. The preoperative diagnosis was multiple HCCs in five patients and combined HCC and ICC in one patient because CoCC lesions were too small to make a definitive diagnosis using various imaging techniques. Both HCCs and CoCCs can be detected at an early stage due to regular check-ups for chronic liver disease. All patients except one had survived without recurrence for a median of 24 months at the time of publication of this case report. The only exception was reported by Suzumura *et al.* [9]. This case developed hepatoduodenal ligament lymph node recurrence, although it was not clear whether the HCC or CoCC was the origin of the recurrence.

**Table 1:** Surgical cases of synchronous double cancers consisting of primary hepatocellular carcinoma and cholangiolocellular carcinoma

Author	Age	Sex	Viral infection	Localization	Size (mm)	Underlying liver disease	Treatment	Prognosis
				CoCC/HCC	CoCC/HCC			
Matsuda *et al*. [5]	70	M	HCV	S7/S4	22/44	Chronic hepatitis	Partial resection	30 months alive without recurrence
Ikeda *et al*. [6]	64	M	HBV	S8c/S8a	22/17	Liver cirrhosis	Segmentectomy	8 months alive without recurrence
Kawano *et al*. [7]	68	F	Negative	S3/S6	9/20	NASH	Partial resection, RFA	41 months alive without recurrence
Sunahara *et al*. [8]	71	M	HCV	S6/S4	8/28	Chronic hepatitis	Partial resection	8 months alive without recurrence
Suzumura *et al*. [9]	45	M	HBV	S7/S6	10/23	Liver cirrhosis	Segmentectomy	20 months died
Our case	58	M	HBV, HCV	S3/S7	8/14	Chronic hepatitis	Partial resection	24 months alive without recurrence

*CoCC* cholangiolocellular carcinoma, *F* female, *HBV* hepatitis B virus, *HCC* hepatocellular carcinoma, *HCV* hepatitis C virus, *M* male, *NASH* non-alcoholic steatohepatitis, *RFA* radiofrequency ablation

When mature epithelial cell compartments of the liver, hepatocytes, and/or cholangiocytes are damaged or inhibited in their replication, hepatic stem/progenitor cells are activated [15, 16]. Hepatic stem/progenitor cells may give rise to various types of primary hepatic cancer, including HCC, ICC, CoCC, and combined HCC and ICC, depending on the stage of differentiation when carcinogenesis occurs. Although the cellular origin of synchronous double primary hepatic cancer is unclear in our case, the inflammatory destruction of liver tissue caused by chronic hepatitis B and/or C virus infections and the consequent repair process may contribute to the different types of primary hepatic cancer (for example, HCC and CoCC) by stimulating both mature hepatocytes and hepatic stem/progenitor cells. Since inflammation may give rise to various combinations of synchronous double primary hepatic cancer in patients with chronic liver disease, it is important that more attention is paid to synchronous double primary hepatic cancer and further cases need to be accumulated.

## Conclusions

We described synchronous double primary hepatic cancers consisting of HCC and CoCC and reviewed the literature.

## Abbreviations

CoCC: Cholangiolocellular carcinoma
CT: Computed tomography
EMA: Epithelial membrane antigen
HCC: Hepatocellular carcinoma
ICC: Intrahepatic cholangiocarcinoma
S3: Segment 3
S7: Segment 7
